# Supplementary material for: The causal relationship between the human gut microbiota and pyogenic arthritis: a Mendelian randomization study
Source: Front Cell Infect Microbiol. 2024 Nov 26;14:1452480. doi: 10.3389/fcimb.2024.1452480 (PMC11629706; doi:10.3389/fcimb.2024.1452480)
Supplement: Supplementary file 1 [file DataSheet1.zip › mendelian test/ebi-a-GCST90027683.csv_forest.pdf]

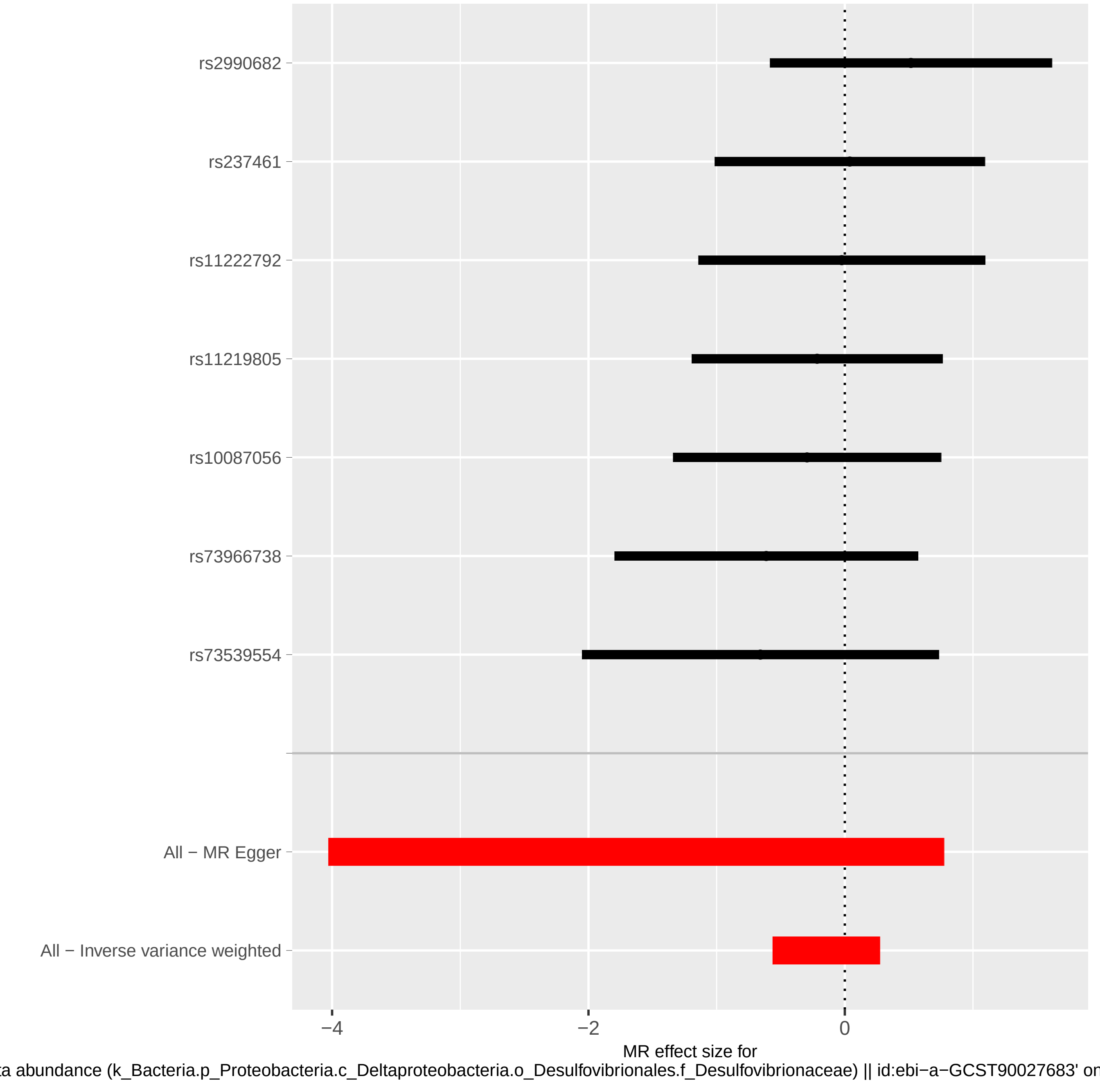

rs2990682
rs237461
rs11222792
rs11219805
rs10087056
rs73966738
rs73539554
All − MR Egger
All − Inverse variance weighted
−4
−2
0
MR effect size for
ta abundance (k_Bacteria.p_Proteobacteria.c_Deltaproteobacteria.o_Desulfovibrionales.f_Desulfovibrionaceae) || id:ebi−a−GCST90027683' on
